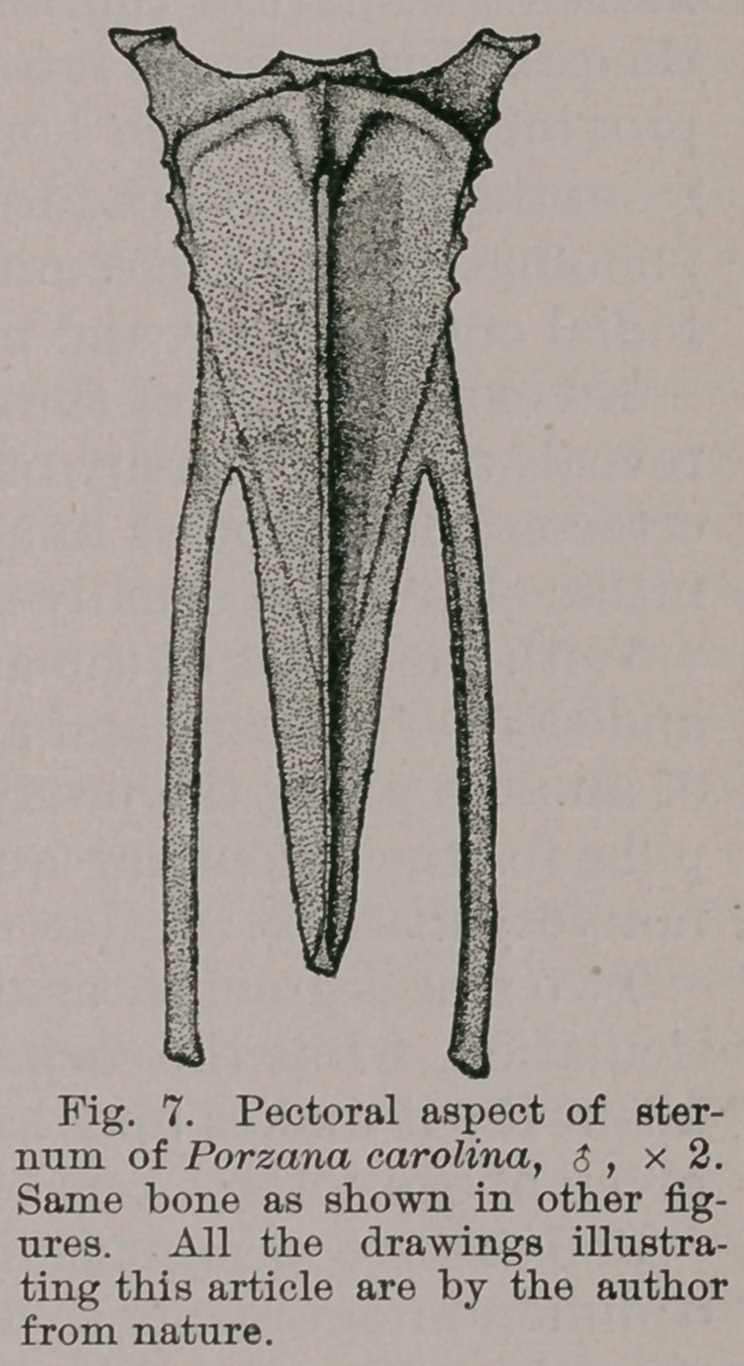# Osteology of Porzana Carolina—Carolina Rail

**Published:** 1888-07

**Authors:** R. W. Shufeldt

**Affiliations:** U. S. A.


### Art. XVII.—OSTEOLOGY OF PORZANA CAROLINA.

### (THE CAROLINA RAIL.)

BY R. W. SHUFELDT, M.D., C.M.Z.S.

As defined by the American Ornithologists' Union, in our Check List, the Order Paludicolæ, containing the Cranes, Rails, etc., is primarily divided into two sub-orders, the (1) Grues or the true Cranes, and (2) the Ralli, containing the Rails, Coots and Gallinules, etc.

The family *Rallidæ* occur in this latter group, wherein the genus *Porzana* is well represented by the subject of the present memoir,—the common Sora or Carolina Rail.

A complete account of the osteology of this ralline form has never been published, and as its skeleton contains many points of interest, to say nothing of importance when we come to compare it with other types, I will devote a few brief observations to it here. When my material better admits of it, it is my intention to quite thoroughly compare the anatomy of our several forms of American Cranes and Rails.

OF THE SKULL :—(Figs. 1, 2 and 3.) Upon lateral view of this part of the skeleton in our Sora Rail, we are to observe the large sub-oval narial aperture that characterizes it, occupying, as it does, nearly the entire area of the side of the superior mandible.

This, taken in connection with the wide interspace between the palatines, anteriorly, on the under aspect of the skull, reduces the premaxilla (*Pmx*) almost to its minimum amount of bony support, and we find its dentary processes long slender limbs, while in the medium line above, the rather narrow culmen is convex, and nearly straight to the tip. Here, for the anterior third of this mandible it slopes uniformly, to the cultrate edges at the side ; the under aspect of this portion being thoroughly concaved.

A *nasal* has its anterior border so sculpt that holorhinalism is most perfectly produced (*n*); while its descending fork anchyloses with the premaxillary and maxillary in a manner most commonly seen among schizognathous birds generally. The lacrymal (*l*) freely articulates with the outer margin of the frontal bone, and somewhat below the plane of its superior surface. It is a very delicate structure in this Rail, consisting of a thin horizontal scale of bone, that projects as a process behind (Fig, 2), and develops a twisted, spiculaform descending process in front, which has its free, lower end in life, well above the maxillary bar. The wing of the mesethmoid, though ample, does not entirely partition the orbital and rhinal cavities. It is of an oblong form, and has its supero-external angle fused with the outer margin of the frontal. A large vacuity absorbs nearly the entire area of the inter-orbital septum, while the apertures on the anterior wall of the brain-case are far larger than is necessary for the mere exit of the nerves that pass out of them, the pair for the first nerves having merged into one foramen.

Very frail, and of nearly uniform calibre, the infra-orbital bar connects the quadrate and maxillary by a gentle curve, where its suture can usually be made out, even in the adult individual.

Either *quadrate* (*q*) has two tranverse facets upon its mandibular foot, the inner one being vertically cleft upon its anterior aspect. Its orbital process is broad and truncate at its extremity. The mastoidal limb is decurved, and supports at its summit two articular heads.

Fig. 1. Right lateral view of the skull of *Porzana carolina*, adult ♂, × 2; *l*, lacrymal; *Pmx*, premaxillary; *Mxp*, maxillo-palatine; *n*, nasal; *pl*, palatine; *pt*, pterygoid; *q*, quadrate. From a specimen in the author's cabinet.

The sphenotic and squamosal processes are but fairly developed, though the valley between them is well marked. Below the latter projection the external osseous ear-conch is very large, and illy supplied by bony surrounding walls for its defence. Viewed upon this aspect, the lateral cranial walls are beautifully rounded and smooth to a fault.

This condition of the external cranial superficies is uniformly extended to its convex vault above, as may be seen in Figure 1, where a superior view of the skull is given. Upon the same aspect we may also see the transverse line of cranio-facial hinge, which is by no means devoid of mobility in our little Carolina Crake. The position of the lacrymals can be best seen in this drawing (*l*).

On the under side of the skull we find the bony palatal structures and other elements arranged as we already know them to be in the true Rails. The maxillo-palatines are separated from each other in the middle line by a notable interval, the convex surfaces that they then present to each other being nearly parallel. Externally, these lamelliform structures are concave throughout, and are supported in their position not only by the customary offshoot from the maxillary, but by their anterior endings, which make firm connections with the inner sides of the nasals and dentary processes of the premaxillary (*Mx*, Fig. 3). Posteriorly, these little boat-shaped affairs are untouched by the neighboring bones.

The lower border of the rostrum (Fig. 3, *R*) is rounded, while in front it is carried to a conspicuous point, the anterior ethmoidal margin being roundly notched and sharp. (Fig. 1.)

The *vomer* (Fig. 3, *v*) is a free bone, having its hinder moiety cleft, forming lateral limbs that articulate with the ascending processes of the palatines, much as we find it in Divers. Above, this element is longitudinally excavated, while a median keel is developed along its under side. Its apex terminates in three minute prongs, a lateral pair, and a mid-process formed by the anterior end of the keel just alluded to in the last sentence.

The short and compressed *pterygoids* (*pt*) meet the palatine heads, the four mutually conjoining to create a longitudinal gutter on their opposite side, to articulate with the rounded and under surface of the sphenoidal rostrum. They are without basi-pterygoidal processes, and they articulate in the usual way with either quadrate.

From this point of meeting of the *palatines* (*pl*) we are enabled to follow, in either one, the line that passes about their rounded postero-external angle, to form the long, straight outer margin of the bone. A palatine also develops a short inner carination or lamina, which is separated from a similar structure on the opposite bone by a still greater interval than we find between the maxillo-palatines. The "ascending processes" of these elements grasp the bar of the rostrum, and proceed forwards to support the vomerine limbs, as already described. They are barely separated in the middle line. The maxillary processes of the palatines are long and slender; they proceed forwards, parallel to one another, to meet the usual struc-

Fig. 2. Upper view of skull of *Porzana carolina*.

Fig. 3. Under view of the same. Both the same specimen as Fig. 1., and both × 2. Lettering as before; *v*, vomer; *Mx*, Maxillary; *R*, Rostrum. The mandible has been removed in each case.

tures at their extremities, in the typical schizognathous articulation (Fig. 3).

Passing to the basi-sphenoid, we find that the bony lip that overhangs the double anterior entrance of the Eustachian tubes, to be short and with rounded edge, not flake-like and sharp, as it is in so many other birds.

Now, posterior to this, all the basal area of the cranium proper, the basi-temporal region, is notably smooth, and withal generally convex. It lacks that sharp definition and angularity due to a more prominent elevation of certain of its features, or in other places a greater depression of them, as we so often find it to be the case in other groups of birds. The comparatively large foramen magnum is roundly cordate in outline, and is nearly in the horizontal plane, while, on the other hand, the condyle is strikingly small, being of a hemispherical form and completely sessile. As we find among the skulls of all vertebrates, I see that in the specimens of skulls of *Porzana* at my hand, some have the condyle relatively much larger than it is in others, while some have the foramen magnum decidedly more cordate in outline. In calling attention to this fact, I mean to say, that birds form no exception to the rule among vertebrates generally, that no two skulls of the same species are alike in all particulars, and it is simply out of the question to present descriptions of *details* in structure that will answer for every specimen. If there could be such a thing as a type skull of *Porzana*, all the skulls we could collect and compare with it would be found to present differences, not only of the kind I have mentioned, but undoubtedly many others. At the same time it must be remembered that the fundamental principles upon which the skull is built for the species, using that word in its present biological acceptation, remain the same. Such principles I have attempted to seize upon for my description of the skull of this Rail, as given above, explicitly stating the exceptions when they have fallen within my observation.

Viewed from above, the *mandible* has the true V-shape, as we might be led to suppose from Fig. 2. The symphysis is short, being scooped out above, and rounded below. Each ramal margin is cultrate along its superior margin, and smoothed off inferiorly. The sides included between them are, for the most part, flat, and nearly vertical. A large ramal vacuity is outlined, and would exist, but in all the specimens examined the splenial element usually succeeds in filling the most of it in.

As in some of the water birds, we find a small foramen in the surangular piece that appears to be constant. I have yet to find a specimen in which the coranoid process is well developed; indeed, its usual site in other birds is here smooth and unbroken.

Coming to the articular cups at the hinder ends of the ramal limbs, they are found to be deep and drawn out into the usual processes towards the mesial plane. At the tip of each of these occurs a single pneumatic foramen.

Huxley lays down the rule that the angle of the mandible in the Geranomorphæ is truncated, and, strictly speaking, this is the case in the Rail we have before us; there is, however, a strong recurved process springing from the postero-external angle of either of these mandibular cups for the quadrates (Fig. 1), that fully merits attention in this connection.

All the elements of the *hyoidean apparatus* are very delicately constructed, the thyro-hyals curving up behind the cranium, are reduced to almost hair-like dimensions, so slender is their calibre. Glosso-hyal and cerato-hyals both remain in cartilage throughout life, and the basi-branchials seem always to be in one piece in the adult bird; the first one being nearly double the length of the second.

*Of the remainder of the Axial Skeleton:* –After carefully counting the vertebræ in the spinal column of *Porzana carolina*, in a sufficient number of specimens, I find that this Rail possesses thirteen vertebræ in the cervical region, wherein in the adult the pleurapophyses co-ossify with the lateral aspect of the centra.

In the fourteenth and fifteenth vertebræ free ribs are found, which are without epipleural appendages, and do not reach the sternum below. Then follow seven more (which includes the twenty-second), that may be considered as the true dorsals. These are all freely movable upon each other, and all have ribs, only the first five pairs of which, however, meet costal ribs below, the remaining two pairs having free hæmapophyses at their lower ends. A pair of rudimentary ribs are also found freely attached to the first sacral vertebræ. Only those ribs which articulate with true costal ribs have epipleural appendages, and these freely articulate with their posterior borders after the manner they do in all birds which do not have them anchylosed at these points.

To form the pelvic sacrum twelve other vertebræ are very thoroughly anchylosed together and fused with the ilia at their sides. Next follow seven movable segments and a pygostyle which constitute the skeleton of the tail of *Porzana.*

Thus we see that the Carolina Crake has forty-one vertebræ in its spinal column, exclusive of the terminal piece, that may be composed of perhaps three or four more.

There is little to be said of the atlas, more than it has the usual ornithic form ; its neurapophyses are somewhat frail, and its articular cup is perforated at its base by the process of the second vertebra.

This latter segment lacks the lateral canals, and develops a large laminate hypapophyses, a feature likewise found upon the third, fourth and fifth vertebræ, where it is very small. A very meagre neural spine is usually found on the first three of these segments after we pass the atlas.

The carotid canal is never closed over, it monopolizing the place of the ventral spines in the sixth to the tenth cervicals inclusive, after which a thin, quadrate ventral spine again takes its place, to appear for the last time on the first dorsal vertebra.

In mid-cervical region the parapophyses are quite long and slender, but they gradually diminish in length as we pass down the series. The postzygapophyses experience a similar change in the same vertebræ, while the prezygapophyses are never very long or prominent anywhere in the column.

Heterocœlous articulation of the centra is the variety found in the *Rallidæ* generally, as it is here.

The dorsal vertebræ have lofty neural spines which interlock with each other at the anterior and posterior angles of their crests above. The centra are broad below, having much the form in each case of the ordinary dice-box, while the diapophyses at their sides are short and not interlaced, either by ossified tendons, nor projecting metapophyses, although these latter are present. Pneumaticity seems to be but partially carried out in any of the vertebræ of this Crake.

Turning to the caudal vertebræ, we are struck with their high neural spines, which are much compressed laterally, and their short transverse processes, these latter being bent downwards to a considerable degree. The pygostyle is of a quadrilateral figure, with rather a thickened apex, and its size is not above that of one of the vertebræ themselves, either in height or depth.

*Porzana* has a pelvis of an unusually interesting form, as I have already had occasion to refer to elsewhere.

Regarding this bone first upon its dorsal aspect, it is to be noted (Fig. 4) that the anterior extremities of the ilia are truncated, and this in such a way that acute externo-lateral angles are created. Posterior to this each ilium, as far back as the acetabulum, is long and narrow, being concave in the longitudinal direction and somewhat so in the vertical one.

The sacrum in the postacetabular region and nearly opposite these rings shows usually three pairs of large interdiapophysial foramina, while the ilia further back present each a subquadrilateral convex surface, that lies nearly in the horizontal plane. These surfaces are produced well behind the sacrum into rather triangular projections, that turn slightly towards the median line, and each other (Fig. 4).

Fig. 4. Dorsal aspect of pelvis of Porzana carolina ♂ + 2.

A side view of the pelvis, (Fig. 5) shows that the neural spines of the leading sacral vertebræ have thoroughly coossified to form a conspicuous crista, which is finisned off along its superior margin by a thickened edge. Now, the antero-internal angles of the ilia nearly meet this latter rim, and from this point the iliac margin dips gracefully downwards making rather a long concave curve, to rise again and arch over the cotyloid ring and ischiac foramen, to pass round behind, forming the boundary of the overhanging ledge that is seen to the rear of these apertures, and finally terminating at the sacrum after surrounding the posterior iliac projection.

The external margin of this pre-acetabular portion of the ilium is much less curved, and it is in the horizontal and not the vertical plane, as is the curve alluded to above. The surface alluded to between these two lines faces nearly directly outwards, and only slightly upwards. *Porzana carolina* has quite a prominent propubis jutting out from its usual site below, and in front of the acetabulum. This latter presents nothing worthy of particular note, while the ischiac vacuity behind it is more of a sub-circular form than we find it in most birds. The ilioischiac area posterior to this orifice is overshadowed by a jutting ledge, developed by the ilium above, much as we find it in *Geococcyx californianus*, though not quite as manifest. A deep triangular notch defines, upon the posterior pelvic border, the landmark between ilium and ischium. This latter element does not meet the rib-like postpubis behind, being separated from it by quite an interval. Nor is the obturator foramen closed by the same means in front, as is often the case.

Fig. 5. Right lateral aspect of pelvis *Porzana carolina*, × 2. Same specimen as shown in other figures.

On the under side of the pelvis, we find the sacral swell to accommodate the enlargement of the myelon, to be very marked, coming out as it does to the very external iliac borders. That vertebra which is opposite the bony division between the cotyloid ring and ischiac foramen, throws out its apophysial struts as lateral pelvic supports at this point.

Posteriorly, reduplication of the ilia forwards, form deep fossæ, one on either side, at the back part of the pelvic basin, these being separated by the last two sacral vertebræ which are specially modified for that purpose.

From my description it will be seen that *Porzana* and *Geococcyx* possess many points in common in their pelvis.

1. Both have the same form of the sacral crista in front.
2. Both have the same pattern, a peculiar one, of the pre-acetabular portion of the ilium.
3. Each has a propubis, and the postpubis does not extend much beyond the ischium behind.
4. In each the ilium develops an overhanging ledge posteriorly, and the ischiac foramen is sub-circular in outline.
5. The ilia extend beyond the sacrum posteriorly, and at the posterior borders behind, a notch shows where these bones join the ischia.

With respect to the propubis, Professor Marsh says, in his work upon the Odontornithes, that "the remnant of the reptilian pubis is still plainly to be seen, especially in *Geococcyx.* It is not improbable that the retention of this process may be due in part to the habits of certain species, as it seems to best developed in running birds, and those that especially use the posterior limbs." (p. 72.)

As the Rails are great runners, too, it would seem that these birds, possessing, as we have seen, almost identically the same style of pelvis as *Geococcyx*, afford us additional examples in support of the correctness of Professor Marsh's conclusions. Whether, however, the form of the pre-acetabular portion of the ilium has anything to do do with this, is a question which at present I am not prepared to answer. In birds that possess it, as we know, the *ambiens* muscle arises from the apex of the propubis, as in *Gallus bankiva.*

From the fact that the postpubis and ischium are well separated after passing the obturator foramen, I would say that the pelvis of *Porzana* was even for a lower order than *Geococcyx*, agreeing as it does in this respect with the pelvis of such ornithic types as *Colymbus*, *Dromœus novæ-hollandiæ* and *Tinamus.*

This question will well repay further investigation, with the view of determining how far such peculiarities are really due to the habits of the bird, and how much depend upon heredity.

Chief among the notable points presented by the *pectoral arch* are the unusually long and narrow blades to the *scapulæ.* In old birds these, not uncommonly, are each more than twice as long as the corresponding coracoid. They taper to points behind, are exceeding narrow and uniformly curved, the convexities being towards each other. The mode of articulation of either one of them is by resting, as usual, upon the scapular process of the coracoid, which, in this Rail, is very wide and having its antero-internal angle used at a *point d'appui* for the head of the clavicle. A *coracoid* is fashioned after a very common form of this bone, as seen among birds at large ; its scapular process is produced for some distance down the side of the shaft, and shows a minute antero-posterior perforation near its middle. The sternal extremity of the coracoid is expanded, as we so often see it in this class, while here its posterior aspect is much concaved. The infero-lateral process is fairly well developed.

The *furcula* has the form of a deep and rather broad U, without the slightest semblance of a hypocleidium below. The clavicular heads of this bone are scarcely at all pronounced ; indeed, this element is comparatively very fraiily constituted, and not much curved in the antero-posterior direction.

I believe, with the exception of certain portions of the skull, and vertebræ beyond the pelvis, the entire skeleton of *Porzana* is a non-pneumatic one.

We find the *sternum* assuming a very quaint form among these short-billed Rails ; it being long and narrow, profoundly cleft, once on either side of its xiphoidal extremity, and with a deep notch occupying the anterior border above the manubrial site (Figs. 6 and 7).

The costal processes are prominent, while the borders of the same name which slope down behind them, bearing on either side the hæmapophysial facets, occupy less than one-fifth the entire length of the lateral border in each case.

Its keel is ample, and extends the full length of the sternal body. In front, its border is concave, and vertically grooved in the median line above, while the lower margin of this part of the bone is convex, and embellished with a raised rim.

Finally, the coracoidal grooves are very long, being

Fig. 6. Right lateral view of sternum of *Porzana carolina*, × 2. From same specimen as before.

Fig. 7. Pectoral aspect of sternum of *Porzana carolina*, ♂, × 2. Same bone as shown in other figures. All the drawings illustrating this article are by the author from nature.

carried clear across the base of either costal process to the lateral border. They do not quite meet in the median line.

Passing to the lateral xiphoidal projections formed by the deep clefts referred to above, we find them to be slightly curved, very long, of a uniform width, and somewhat enlarged at their ends.

The middle part of this hinder portion of the sternum has the form of an isosceles triangle with a short base, and its apex pointed to the rear, being at the same time within the imaginary line joining the ends of the lateral xiphoidal limbs.

Were this part of the sternum entire, then on its dorsal aspect we should have quite a concavity presented us, and this is the case with that part of the bone lying between the costal processes and in front of the aforesaid clefts of its hinder extremity.

Of the Appendicular Skeleton :—In an adult specimen of *Porzana carolina* I find the humerus to be 3.6 cms. long ; the ulna 3, and the pinion 3.7 cms. long. A slight curve marks the shaft of the *humerus* about its middle, and on the radial side where it is convex. The ulnar crest at the proximal end of the bone is prominent, being separated from the humeral head by a deep notch, while the pseudo-pneumatic fossa it circumscribes is notably shallow. The radial crest is short, and not very striking.

An examination of the distal extremity of the humerus reveals nothing worthy of especial remark. It has a small ectocondyloid process in addition to the other common ornithic characters usually seen there.

With the bones of the antibrachium, we have a straight and slender *radius*, and an *ulna* that exhibits considerable of an arch along the proximal third of its shaft. The papillæ for the secondary quill feathers are present, though not very distinct.

Two free segments, as usual, are found in the carpal articulation, while the *carpo-metacarpus* has nothing peculiar about it ; it makes up 2.1 cms. of the length of the pinion given above.

Pollex digit terminates in a free claw, which pierces the common integuments to appear externally, where it is encased by a sheath of horn like the ungual phalanges of pes.

No such appendage is to be found at the last joint of index digit, while the proximal joint of this finger has its expanded portion rather narrow and composed of one unbroken piece of bone. Mid-metacarpal supports its usual small finger-joint.

In the pelvic extremity we have a femur 3.85, a tibiotarsus of 6, a tarsometatarsus of 3.8, and the middle toe of pes, 4.65 centimetres long.

The *femur* develops a trochanterian ridge that rears above the articular surface of the summit; the femoral head, however, is small, though prominent, and has a well defined pitlet for the insertion of the round ligament, not diffuse as it is in some birds.

Its shaft is somewhat slender, slightly bowed to the front along its continuity, terminating in the usual condylar prominences below. Of these, the inner one is the rather more manifest in front, but the lowest point of either seems to lie in the same horizontal plane. The channel separating them anteriorly subsides on the shaft opposite their own endings.

A *patella* does not develop in *Porzana carolina*, its substitute being seen in a thick, cartilaginous deposit in the tendon, at its usual site.

In the *tibio-tarsus*, the cnemial crest is produced well above the articular summit of the bone, and the two ridges which embellish its anterior portion below the procnemial ridge, is far the more conspicuous, jutting out directly to the front, but soon terminating abruptly below. The ecto-cnemial ridge is smaller, is in a plane parallel to the fibular ridge, and terminates in a decurved process or hook opposite, and in front of the head of the fibula.

As for the shaft, we find it nearly straight, smooth, but inclined to become somewhat four-sided immediately before expanding into its condylar-distal extremity. This part exhibits the usual longitudinal groove in front, crossed by the bridge of bone for the extensor tendons. The inter-condylar notch is very deep in front, broad, and rather shallow behind. And of the two condyles, the outside one is considerably the broader and larger, while but little difference is to be noticed between them upon posterior aspect.

The lower, and extremely delicate end of the *fibula* fuses with the tibio-tarsal shaft at about its middle, though we occasionally meet with specimens wherein it may be traced still lower down. Its head, above, has the usual form, but is notably compressed in the lateral direction.

A fairly well developed hypo-tarsus is found at the upper and back part of the next lower segment of this Rail's leg, —*the tarso-metatarsus.* It consists of a median longitudinal ridge, flanked on either side by a shorter, parallel, and less prominent one. This, of course, gives rise to two grooves, for the guidance of the tendons, and none of the latter perforate the body of this process as we often see in many other birds. The sides of the shaft of this bone are more or less flat and slightly twisted below, from without towards the inner side, or median plane. A decided fossa occurs at the upper and anterior end of the shaft, which grows shallower lower down, to gradually disappear at the junction of the upper and lower thirds.

Of the trochleæ at the distal extremity, we find that the middle and lowest one is sharply separated from the outer and next highest one, by a deep cleft, the arterial foramen occurring in its groove above. The inner trochlea is turned considerably to the rear, while above it, suspended in the usual manner by ligament, is seen rather a large accessory metatarsal, giving support to a strong hallux, composed of basal joint and claw.

The phalanges of the anterior podal digits are long and slender, each terminating in a well-developed ungual joint. These latter are laterally compressed and show some degree of curvature in the longitudinal direction.

For the inner two toes the basal joints are the longest and largest in calibre, while they progressively decrease in both these respects, from joint to joint, as we go towards their nails. This obtains also in the inner toe ; but here we find that, counting the ungual joint as one, the penultimate phalanx is somewhat longer, though it is not as large in calibre as the joint that precedes it.

Brief Recapitulation of the Skeletal Characters of Porzana carolina.—1. External narial apertures on sides of superior osseous mandible very large.

2. Lacrymal bones free, and possessed of delicate descending processes.

3. Pars plana ample, quadrilateral in outline, supero-external angle fusing with underside of frontal bone.

4. Vacuity in inter-orbital septum very large.

5. Mastoidal head and mandibular foot of quadrate each have two articular prominences.

6. Vomer free, deeply cleft behind for the rostrum, minutely tricorunate at its anterior apex.

7. Maxillo-palatines concavo-convex, long, and separated from each other in the median line.

8. Postero-external angles of palatines completely rounded off. Their maxillary processes long and very slender, being widely apart and nearly parallel to each other.

9. Pterygoids rather short, and lamelliform.

10. Mandibular angles of mandible truncate below, but developing recurved processes immediately behind the articular cups.

11. Occipital condyle small, foramen magnum relatively very large.

12. Type of skull holorhinal and schizognathous.

13. The furcula U-shaped, deep (and as a negative character) without hypocleidium. Scapulæ long and narrow, coracoids rather short in comparison.

14. Sternum with lofty costal processes ; notched anterior border, rudimentary manubrium, xiphoidal extremity profoundly cleft once on either side ; carina deep, extending entire length of body, which latter, seen from above, is long and narrow.

15. Length of pelvis far exceeds its greatest width. Supero-internal margins of preacetabular portions of ilia concave, exposing on lateral view the fused neural spines of sacral vertebræ. Propubis present. Ischiac foramen subcircular. Postacetabular portion of ilium develops an overhanging ledge of bone (as in *Geococcyx*). Postpubis separated from ischium for its entire length after passing the obturator foramen. Ilio-ischiac notch present on posterior margin of bone. Ilia extend well beyond the sacrum behind.

16. Pollex digit of manus supports a claw.

17. Cnemial crest of *tibio-tarsus* rises above articular summit of the shaft. Bony bridge for extensor tendons at distal end of the bone, thrown directly across the groove. External condyle below it, much larger than the internal one, on anterior aspect. Hypotarsus of *tarso-metatarsus*

grooved only ; mid-trochlea the lowest, inner one the highest on the shaft, and is turned to the rear. Arrangement of phalanges of podal digits normal (2.3.4.5), the points long and slender, but on the whole harmoniously proportioned as to lengths and calibres.

## Figures and Tables

**Fig. 1. f1:**
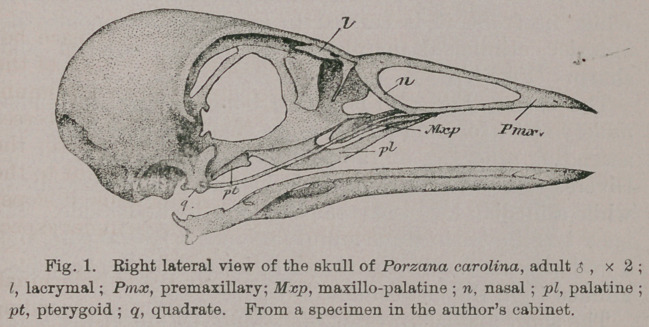


**Fig. 2. Fig. 3. f2:**
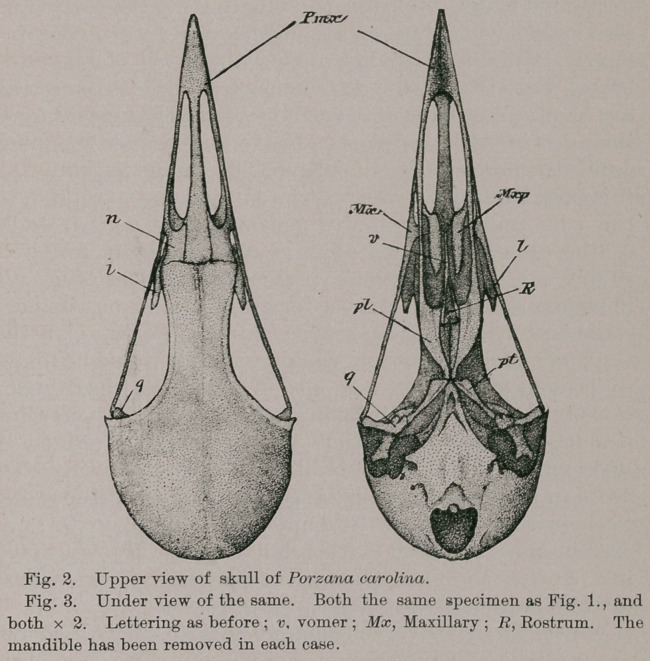


**Fig. 4. f3:**
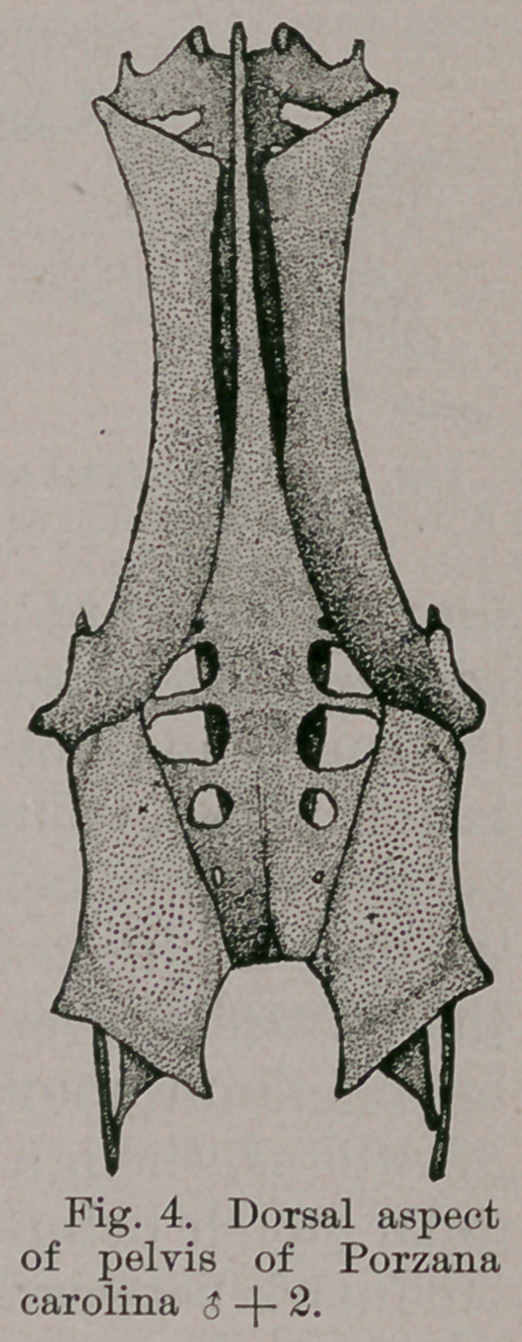


**Fig. 5. f4:**
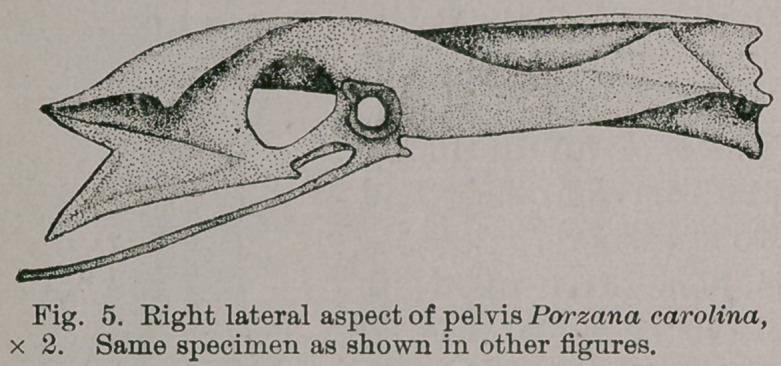


**Fig. 6. f5:**
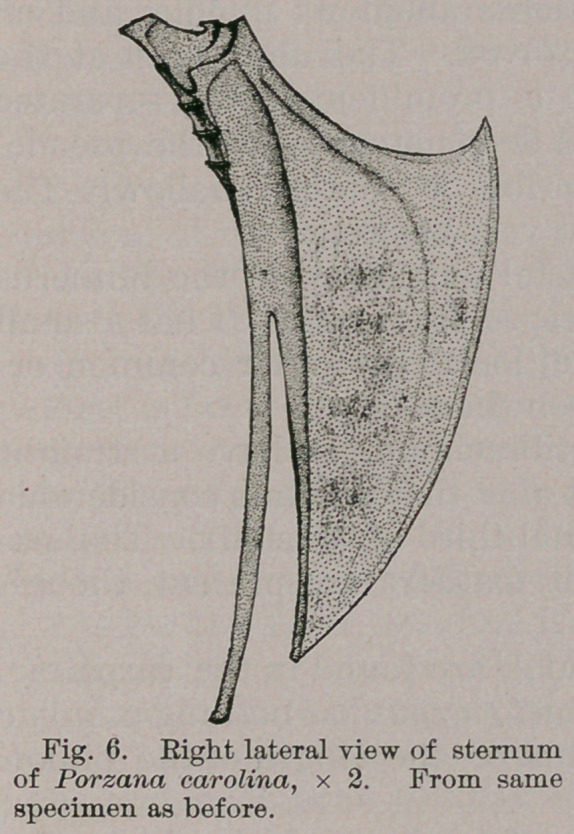


**Fig. 7. f6:**